# Spondylo-Thoracic Dysplasia: Survival, a Rare Occurrence

**DOI:** 10.7759/cureus.74894

**Published:** 2024-12-01

**Authors:** Upendra P Sahu, Rufeena J, Suman Kumar, Nimisha Vatsana

**Affiliations:** 1 Pediatric Medicine, Rajendra Institute of Medical Sciences, Ranchi, IND

**Keywords:** crab rib deformity, intestinal herniation, recurrent pregnancy loss, respiratory distress, spinal dysraphism, spondylo-thoracic dysplasia, volume depletion patho-anatomy

## Abstract

Spondylo-thoracic dysplasia (STD) is a set of rare congenital abnormalities involving the vertebrae and thorax, leading to significant morbidity and mortality due to respiratory insufficiency and associated anomalies. Clinically, neonates present with scoliosis, vertebral segmentation defects, and severe respiratory compromise, resulting in early neonatal death. These children have a unique patho-anatomy of volume depletion deformity of the thorax, resulting in thoracic insufficiency. Hence, long-term complications such as restrictive lung disease, recurrent chest infections, and pulmonary hypertension need to be meticulously assessed.

We report the case of a one-day-old newborn, a term male baby, who had respiratory distress and swelling over the left lower chest since birth. Clinical examination revealed a soft, non-pulsatile, compressible, and reducible swelling on the left hypochondrium and fine crepitations in bilateral basal lung fields. The baby had a short neck with an upper segment to lower segment ratio of 1:2.7 and scoliosis. Investigations including a chest X-ray showed crab rib deformity with scoliosis, displacement of the left lower ribs, and fusion of spondylo-costal joints. Ultrasound of the abdomen revealed a left lateral abdominal wall defect and herniation of bowel loops posterior to the spleen. Computed tomography (CT) of the thorax and abdomen showed ground-glass opacities in bilateral lung fields, right mediastinal shift, hemivertebrae of D6, D7, and D8, a bifid eighth vertebral head, an absent left eighth rib, and spina bifida of L4 to S5 vertebrae. A clinico-radiological diagnosis of STD was made. The baby was managed conservatively, discharged, and was followed up routinely until the age of one year.

Our case is one of the few variants of STD with an absent rib, post-splenic intestinal herniation, classical crab rib deformity, hemivertebrae, and spina bifida that has survived the neonatal period and infancy, thus providing an opportunity for further understanding and treatment of the disease process through future follow-ups. To the best of our knowledge, our case is the first case of STD from Eastern India to survive infancy with these features. Early recognition, workup, and follow-up of these babies are essential to monitor growth patterns and associated comorbidities. Hence, any case of congenital deformity with recurrent pregnancy loss should undergo complete clinico-radiological evaluation with genetic counseling and long-term monitoring for prompt management of associated morbidities.

## Introduction

Spondylo-thoracic dysplasia (STD) is a set of rare congenital abnormalities involving the vertebrae and thorax, leading to significant morbidity and mortality due to respiratory insufficiency and associated anomalies [1]. STD was previously grouped under Jarcho-Levine syndrome, which consists of spondylo-costal and spondylo-thoracic types. Although initially considered to be lethal, recently documented milder forms have been found to be compatible with life [1]. Clinically, neonates with STD present with scoliosis, vertebral segmentation defects, and severe respiratory compromise, resulting in early neonatal death. These children have a unique patho-anatomy of volume depletion deformity of the thorax, which results in thoracic insufficiency and leads to poor pulmonary clearance. Consequently, these children suffer from long-term complications such as restrictive lung disease, recurrent chest infections, and pulmonary hypertension [2]. The vertebral segmentation defects include spina bifida, spina bifida occulta, myelocele, meningomyelocele, and hemivertebrae. They also exhibit associated abnormalities such as facial dysmorphisms, low-set ears, a short upper segment, umbilical hernias, urogenital abnormalities, and complex congenital heart diseases [3]. Therefore, early detection and regular monitoring of these complications are essential for the timely management of illness and to prolong the life span of these children.

## Case presentation

**Figure 1:**
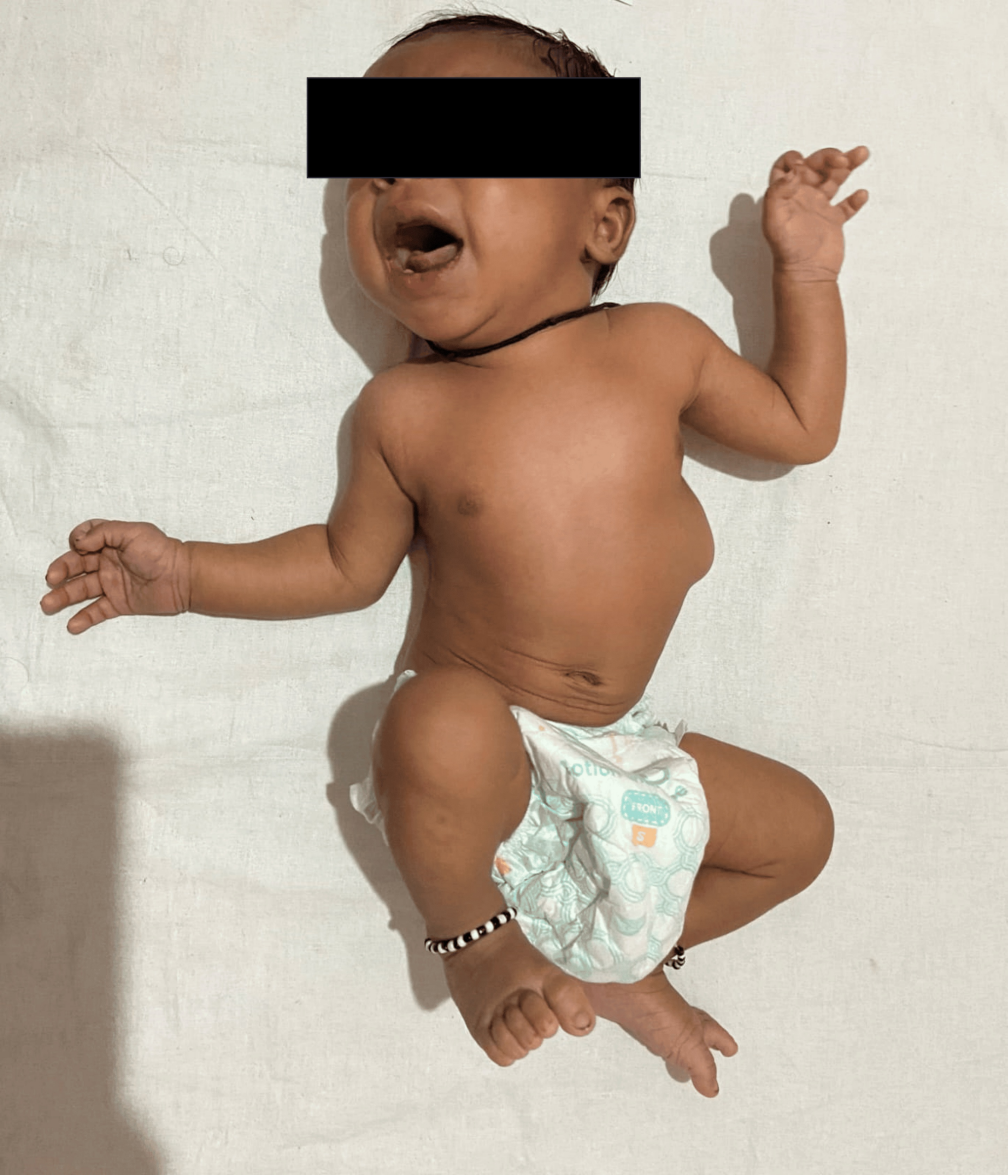
Clinical image of the neonate with spondylo-thoracic dysplasia. The image displays a short neck, malformed chest, and abnormal spinal curvature. There is an abnormality in stature characterized by an asymmetric thoracic cage and scoliotic spine, with a protuberant lump on the left side.

We report the case of an inborn, singleton, term, male baby born by normal vaginal delivery. He cried at birth with Appearance, Pulse, Grimace, Activity, and Respiration (APGAR) scores of 8/10 at 1 minute and 9/10 at 5 minutes, respectively, and a birth weight of 3.5 kg. His mother, a non-consanguinously married 36-year-old, Gravida 3, Para 1, Living 1, Abortion 2 (G3P1L1A2), had normal prenatal ultrasounds and two spontaneous first-trimester abortions. At birth, the baby presented with respiratory distress and swelling over the left lower chest. Clinical examination revealed tachypnea, chest retractions with a Silverman-Anderson Score (SAS) of 4/10, scoliosis, and a soft, non-pulsatile, compressible, reducible swelling on the left lateral chest measuring 4x4 cm. Fine crepitations were noted in bilateral basal lung fields with palpable bony fragments in the left lumbar area. The baby had a short upper segment, with an upper segment to lower segment ratio of 1:2.7. Other anthropometric parameters were normal, with a length of 48 cm and head circumference of 35 cm. The baby was admitted to the NICU for management of respiratory distress. The clinical image of the neonate is represented in Figure 1 below.

**Figure 2:**
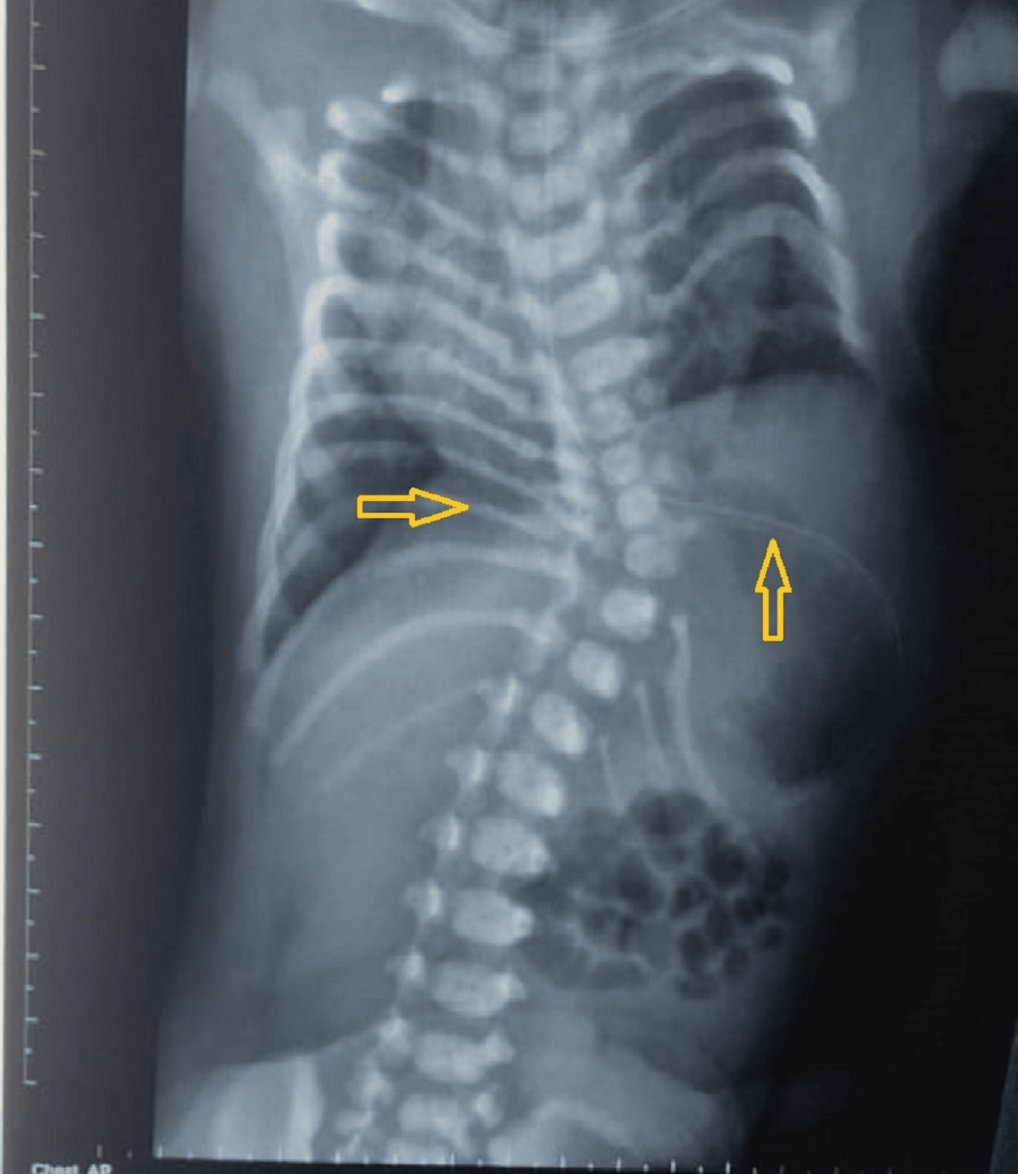
X-ray chest AP view of a neonate with spondylo-thoracic dysplasia. Shows scoliosis, displacement of left lower ribs, and fusion of the spondylo-costal joint, crab deformity. This pathognomonic feature arises from posterior fusion and anterior flaring of the ribs, giving rise to the typical crab-rib deformity [3]. AP: Antero-posterior.

**Figure 3:**
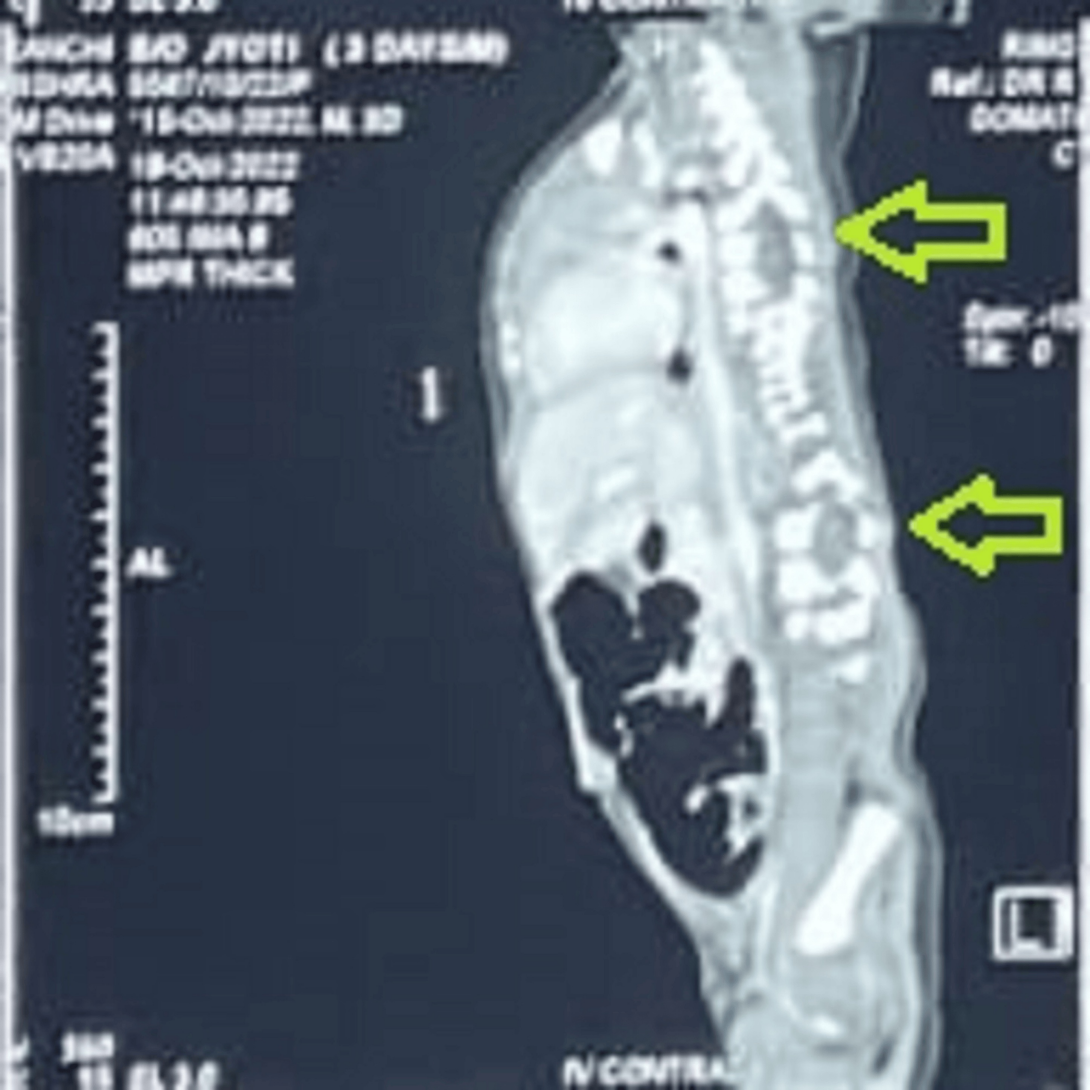
Computed tomography of the thorax and abdomen of a neonate with spondylo-thoracic dysplasia. Shows ground-glass opacities in bilateral lung fields with a right mediastinal shift, hemivertebrae of D6, D7, and D8, a bifid 8th vertebral head, an absent left 8th rib, and L4-S5 spina bifida.

Investigations included a chest X-ray, as shown in Figure 2, which revealed scoliosis, displacement of left lower ribs, and fusion of spondylo-costal joint giving the typical appearance of a crab deformity of the ribs. An ultrasound of the abdomen showed an abdominal wall defect on the left lateral side with herniation of bowel loops posterior to the spleen, which was identified during the clinical examination as a soft compressible swelling. A CT scan of the thorax and abdomen, shown in Figure 3, revealed ground-glass opacities in bilateral lung fields, right mediastinal shift, hemivertebrae of D6, D7, and D8, a bifid 8th vertebral head, an absent left 8th rib, and L4-S5 spina bifida. A 2D echocardiography performed as part of the workup revealed mild pericardial effusion.

The baby was admitted to the NICU with a heart rate of 158 beats/min and a respiratory rate of 90 breaths/min, with an SAS score of 4/10, saturation of 90% in room air, and a capillary refill time of less than 3 seconds. He was managed with oxygen support via bubble CPAP on day 1 of life with a positive end-expiratory pressure (PEEP) of 6 cm of water and an fraction of inspired oxygen (FiO2) of 35%, along with orogastric (OG) tube feeding. By day 3 of life, exclusive breastfeeding was established, and chest physiotherapy was initiated. The respiratory distress improved, and by day 6 of life, the baby was weaned off oxygen therapy. Blood biochemistry showed normal renal function and normal blood gas analysis. Blood culture reports were negative. MRI of the brain and genetic testing were refused by the parents. The baby was hemodynamically stable and was vaccinated and discharged from the hospital on day 8 of life. On follow-up at 1 month, 6 months, and 1 year of postnatal age, the baby was stable, had normal anthropometry, adequate weight gain, and no hospitalizations. Genetic counseling was done, and he was referred to a higher center for surgical management.

## Discussion

STD is a rare congenital disorder affecting the spine and thorax. Clinically, neonates present with scoliosis, vertebral segmentation defects, and severe respiratory compromise, resulting in early neonatal death. STD was previously categorized under Jarcho-Levin syndrome. It was first described in the literature by Jarcho and Levin in 1938, among cases of thoracic insufficiency due to vertebral and limb anomalies [3]. About 400 cases of Jarcho-Levin syndrome have been reported globally, with only 15 cases reported in the Indian literature [4]. Most of these cases have either been misdiagnosed or undiagnosed [5].

Jarcho-Levin syndrome is a congenital skeletal disorder with an autosomal recessive inheritance [6]. Disorders under Jarcho-Levin syndrome have been categorized into two distinct genetic disorders: STD and spondylo-costal dysostosis (SCD) [1, 7].

These children have a unique patho-anatomy of volume depletion deformity of the thorax, resulting in thoracic insufficiency. Hence, long-term complications such as restrictive lung disease, recurrent chest infections, and pulmonary hypertension [8] need to be meticulously assessed. Although genetic studies can confirm the diagnosis with mutations in the MESP, DLL3, LFNG, and HES7 genes, they are not detected in 50% of cases [9]. Therefore, it is primarily a clinico-radiological diagnosis [2]. Very few cases of mild variants have been reported that are compatible with life [10].

With expertise, prenatal diagnosis of STD can be performed by looking for a shortened spine, disorganization of the vertebral bodies, and posterior fusion of the ribs in antenatal scans [9]. If anticipated, specialized neonatal care may improve survival.

In our case, despite a normal antenatal ultrasound, the diagnosis of STD was made postnatally by clinico-radiological assessment. Our case is one of the few variants of STD with an absent rib, post-splenic intestinal herniation, classical crab rib deformity, hemivertebrae, and spina bifida that has survived the neonatal period, thus providing us an opportunity for further understanding and treatment of the disease process through future follow-up.

## Conclusions

Although most of the reported cases have severe respiratory insufficiency, our case represents a rare variant with mild respiratory distress, an absent rib, hernia, and neural tube defects. Only 15 cases have been reported from India, and this is the first case from Eastern India to survive into infancy with these features. According to our research, it is the second case of STD to be reported with post-splenic herniation. Therefore, any case of congenital deformity accompanied by recurrent pregnancy loss should undergo a complete clinico-radiological evaluation, along with genetic counseling and long-term monitoring, for the prompt management of associated morbidities.
